# Deep Learning-Based Human Activity Real-Time Recognition for Pedestrian Navigation

**DOI:** 10.3390/s20092574

**Published:** 2020-04-30

**Authors:** Junhua Ye, Xin Li, Xiangdong Zhang, Qin Zhang, Wu Chen

**Affiliations:** 1 College of Geology Engineering and Geomantic, Chang’an University, Xi’an 710054, China; junhua2009@chd.edu.cn (J.Y.); xiangdong2018@chd.edu.cn (X.Z.); dczhangq@chd.edu.cn (Q.Z.); 2 Department of Land Surveying and Geo-Informatics, Hong Kong Polytechnic University, Hong Kong 999077, China; wu.chen@polyu.edu.hk

**Keywords:** LSTM, CNN, tensorflow, deep learning, pedestrian navigation

## Abstract

Several pedestrian navigation solutions have been proposed to date, and most of them are based on smartphones. Real-time recognition of pedestrian mode and smartphone posture is a key issue in navigation. Traditional ML (Machine Learning) classification methods have drawbacks, such as insufficient recognition accuracy and poor timing. This paper presents a real-time recognition scheme for comprehensive human activities, and this scheme combines deep learning algorithms and MEMS (Micro-Electro-Mechanical System) sensors’ measurements. In this study, we performed four main experiments, namely pedestrian motion mode recognition, smartphone posture recognition, real-time comprehensive pedestrian activity recognition, and pedestrian navigation. In the procedure of recognition, we designed and trained deep learning models using LSTM (Long Short-Term Memory) and CNN (Convolutional Neural Network) networks based on Tensorflow framework. The accuracy of traditional ML classification methods was also used for comparison. Test results show that the accuracy of motion mode recognition was improved from 89.9%, which was the highest accuracy and obtained by SVM (Support Vector Machine), to 90.74% (LSTM) and 91.92% (CNN); the accuracy of smartphone posture recognition was improved from 81.60%, which is the highest accuracy and obtained by NN (Neural Network), to 93.69% (LSTM) and 95.55% (CNN). We give a model transformation procedure based on the trained CNN network model, and then obtain the converted .tflite model, which can be run in Android devices for real-time recognition. Real-time recognition experiments were performed in multiple scenes, a recognition model trained by the CNN network was deployed in a Huawei Mate20 smartphone, and the five most used pedestrian activities were designed and verified. The overall accuracy was up to 89.39%. Overall, the improvement of recognition capability based on deep learning algorithms was significant. Therefore, the solution was helpful to recognize comprehensive pedestrian activities during navigation. On the basis of the trained model, a navigation test was performed; mean bias was reduced by more than 1.1 m. Accordingly, the positioning accuracy was improved obviously, which is meaningful to apply DL in the area of pedestrian navigation to make improvements.

## 1. Introduction

Smartphones are widely used at present, some products of IOT (Internet of Things) have been developed, and many mobile phone applications have been provided in Google Play or Apple Store [1]. Navigation applications and location-based services are now becoming standard features in smartphones [2]. Positioning is a key issue to resolve the business requirements of these products. GNSS (Global Navigation Satellite System) can usually provide good positioning accuracy in an open-sky environment using professional devices; however, the capability of GNSS positioning is degraded in indoor or harsh environments due to signal blockage and multipath. In recent years, pedestrian navigation based on smartphones has developed rapidly. Smartphones are not professional positioning devices; they contain low-accuracy positioning sensors, chips, and antenna. Thus, their positioning capability worsens, especially in a harsh environment. Accordingly, standard GNSS-INS integration algorithms are not applicable and should develop more accurate navigation algorithms in different scenes and stages. Pedestrian activity recognition is vital in the procedure of pedestrian navigation [3,4,5]. With the explosive growth of the capabilities in smartphones, various sensors, such as accelerometers, gyroscope, magnetometers, and barometers, are embedded in smartphones [5]. Pedestrian activity recognition, which utilizes these powerful sensors to recognize different activities, has been gaining considerable attention in recent years [6]. Pedestrian activity recognition is a classification problem; several related methods have been developed in previous studies, such as RF (Random Forest), DT (Decision Tree), and kNN (k-Nearest Neighbor). Pedestrian activity recognition is significant in several fields, such as elderly monitoring and transportation modes recognition, because it can help reduce workload and understand people’s behaviors [3]. In the process of pedestrian navigation, error accumulation is inevitable; diminishing the cumulative error effectively is a challenge for localization systems but is important, because decreased cumulative error improves the localization accuracy [7,8]. In recent years, motion mode recognition can also be used in pedestrian localization system to adjust positioning algorithm to reduce accumulative error. The feasibility of activity recognition for pedestrian localization has been demonstrated by multiple authors [9,10,11]. Therefore, studying of activity recognition is important to improve the effect of pedestrian localization [6].

State-of-the-art solutions for pedestrian activity recognition can be divided into two categories: traditional methods and deep learning-based approaches [12,13]. Traditional methods usually consist of two parts: feature extraction and classification. They rely on extracting complex hand-crafted features, which are laborious and inefficient; thus, they might lead to incapability of real-time identification of pedestrian activities [14,15]. Deep learning methods overcome this shortcoming by fusing the two steps with an NN to automatically learn proper features [6]. Deep learning techniques have revolutionized ML algorithms and their applications [16]. They are used in pattern recognition, natural language processing, and speech recognition. Several deep learning network models have been designed, such as CNN and LSTM, and more and more advanced, complicated deep learning networks will be raised.

Pedestrian motion mode recognition is based on motion sensors that are embedded in mobile devices [17]. In traditional methods, data are processed by various steps, such as preprocessing, data segmentation, extraction of salient and discriminative features, and finally classification of activity [18]. For example, Fan, L. et al. used DT to conduct human activity recognition by these steps [19]. Akhavian, R. et al. combined mobile sensors and machine learning classifiers to construct equipment activity recognition [20]. In deep learning, CNN is widely used for sequential data analysis, and the key to its success lies in the use of convolutional filter hierarchies that consecutively extract feature representations of increasing complexity from raw sensor measurements [21]. However, CNN can only be used for this kind of data by using a sliding-window that carves out consecutive sample data; in this way, the time-series are assumed static and are thus analyzable using CNN [16]. Researchers now use sequential deep learning models. LSTM models are particularly attractive because their specialized internal structure implements a memory that includes a forget function to effectively and selectively focus on those sensory data that are relevant to the recognition process [16].

The rapid development of deep learning has facilitated the use of deep learning algorithms in recognition. Previous researchers have conducted studies on pedestrian activity recognition, for example Bayat, A. et al. used smartphone accelerometer measurement to recognize pedestrian activities [22]. Altun, K. et al. performed localization simultaneously with activity recognition and switched to different contexts according to the detected activities [23]. Guo, J. et al. used a self-learning scheme to monitor patients’ activities by smartphones [24]. Kwon, Y. et al. gave a human activity recognition solution using unsupervised learning with smartphone sensors [25]. Ignatov, A. et al. used CNN and accelerometer data to recognize human activities in real-time [26]. Wang, K. et al. used re-configurable convolutional neural networks for 3D human activity recognition [27]. M. Zeng et al. designed an LSTM network to recognize pedestrian activities [28]. Hassan, M. M.  et al. combined CNN and LSTM networks to recognize pedestrian activities [29]. F.J.O. Morales et al.utilized a deep belief network model for successful human activity recognition [30]. Hassan, M. M. et al. presented a solution to recognize pedestrian activity using deep learning methods and compared it with SVM and ANN (Artificial Neural Network); the proposed solution outperforms the two other methods [31]. Jiang and Yin proposed a method of constructing a novel activity image for DCNN (Deep Convolutional Neural Network) using the gyroscope, total acceleration, and linear acceleration signals [32]. Alskeikn et al. tested the activity recognition performance of DBN (Deep Belief Network) using different parameter settings [33]. Based on pedestrian activity recognition, researchers have attempted to optimize navigation algorithms; e.g., Niitsoo. A. et al. used LSTM network to train sensors’ measurements to estimate step length [34]. Arne Niitsoo et. al presented a solution to estimate the position of mobile objects directly from the raw channel impulse response using deep learning [14]. Wang, Q., proposed a solution to estimate the pedestrian walking distance based on smartphone mode recognition [34].

Although previous studies have presented several pedestrian activity recognition strategies, most of these approaches are immature, especially in a harsh environment combined with complicated movement. Thus, this paper presents a strategy using deep learning algorithms to conduct real-time recognition and adjust and optimize pedestrian navigation algorithms to improve positioning accuracy. The contributions of this study are summarized as follows.

(1) This paper summarizes MEMS measurement preprocessing strategies, including de-noising filtering algorithms, FFT, posture transformation, body acceleration extraction from total acceleration, and feature extraction from measurements. Traditional ML classification methods and commonly used deep learning methods are reviewed, and recognition evaluation methods and navigation update algorithms are presented.
(2) Four main experiments were performed, namely pedestrian motion mode recognition, smartphone posture recognition, real-time comprehensive pedestrian activity recognition, and pedestrian navigation experiment. Seven traditional ML classification methods and two deep learning methods were used in the procedure of recognition. Several test results are presented for comparison and analysis in this paper.
(3) We designed deep learning models using LSTM network and CNN network. On the basis of the trained models, we converted the trained model to a lightweight model, which can be run in Android smartphones. This way provides the possibility to recognize pedestrian activity in real-time.
(4) Real-time recognition was performed in a Huawei Mate20 smartphone. Five comprehensive pedestrian postures, frequently used in pedestrian navigation, were designed. Test results show that the right recognition rate was up to 89.39%, which was useful to enhance the optimization of pedestrian navigation algorithms.

The paper is organized as follows. Section 1 reviews recent studies and development in these aspects of pedestrian activity recognition, traditional ML classification methods, and deep learning algorithms. Section 2 presents the methodology, including measurement de-noising algorithms, horizontal transformation, FFT, body acceleration extraction, ML classification methods, deep learning algorithms, trained model transformation procedure, recognition evaluation algorithms, and pedestrian positioning update algorithms. Four main experiments are summarized in Section 3, namely motion mode recognition using the UCI (University of California Irvine) open dataset, smartphone postures classification using seven traditional ML classification methods and two deep learning modes constructed by LSTM network and CNN. Besides, a real-time test of comprehensive pedestrian activities was done, on the basis of the trained model; the pedestrian navigation experiment was performed at Hong Kong Polytechnic University. Section 4 provides the discussions and analysis of these experiments. Section 5 elaborates the conclusions.

## 2. Methodology

### 2.1. General Procedure of Recognition

**Figure 1:**
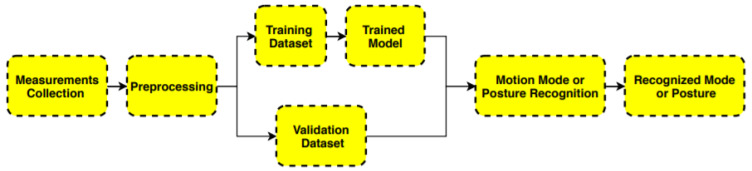
Procedure of recognition.

Recognition involves several steps. In this study, raw measurements of smartphone sensors (accelerometer, gyroscope, magnetometer, etc.) were collected first. Then, these measurements were processed to obtain a useful recognition model. Figure 1 presents the whole workflow of motion mode or smartphone posture recognition for pedestrian navigation.

(1) Collect measurements from smartphone MEMS sensors.
(2) Preprocess sensor measurements and divide the data to training and validation dataset.
(3) Train and optimize the model, and conduct precision test.
(4) Perform prediction.

### 2.2. Preprocessing of Measurements

#### 2.2.1. De-Noising

De-noising can be realized using different methods, including low pass filtering, which removes high-frequency noise. However, low-pass filtering removes high-frequency dynamics that may describe the motion [35]. The methods of de-noising or noise reduction used in the study is discussed as follows:

(1) Digital low-pass filter: Use a conventional low-pass filter with a pre-defined cut-off frequency. A low-pass filter is a circuit that can be designed to modify, reshape, or reject all unwanted high frequencies of an electrical signal and accept or pass only those signals wanted by the circuits designer. In this study, we used Butterworth low-pass filter. The related formula is given as follows:
  (1) Y(n)=a×X(n)+(1−a)×Y(n−1),
  where X(n) is the input signal, Y(n−1) is the filtered signal in last epoch, and *a* is the low-pass filter coefficient.
(2) Mean filtering: Use a moving average window replacing each data element with the mean of the group of data elements before and after it.
(3) Median filtering: Use a moving average window but return the median rather than the mean. The median filter provides a nonlinear approach to filter that can be extremely effective in combating impulse noise with ease of implementation. It does not require multiplication or addition but needs only a fairly quick sorting after each sample. It creates some artifacts, particularly clipping. However, these artifacts are tolerable for most applications [35]. The formula is expressed as follows:
  (2) y(i)=Med{x(i−N),⋯,x(i),⋯,x(i+N)},
  where x(i) is the input signal and y(i) is the filtered signal.

#### 2.2.2. Fast Fourier Transform

A fast Fourier transform (FFT) is an algorithm that computes the discrete Fourier transform (DFT) of a sequence, or its inverse (IDFT). Fourier analysis converts a signal from its original domain (often time or space) to a representation in the frequency domain and vice versa [36]. The DFT is obtained by decomposing a sequence of values into components of different frequencies [2]. This operation is useful in many fields, but computing it directly from the definition is often too slow to be practical. An FFT rapidly computes such transformations by factorizing the DFT matrix into a product of sparse (mostly zero) factors [37].

#### 2.2.3. Transforming Measurements to Horizontal

An advanced step to calculate the norm of acceleration or rotation measurements is to level them to a frame of reference, which usually is the local-level frame (i.e., a frame where the vertical axis is parallel to gravitational force and the horizontal plane is parallel to the Earth’s ellipsoid) [38]. Mizell deduced that, in pedestrian activities, or for low-acceleration activities, the low-frequency component of acceleration measurements can be an estimate of the gravity vector. Therefore, the low-frequency component can be calculated as the average of the acceleration vector:

(3) $${\vec{a}}_{ave}=\mathrm{mean}\left(\vec{a}\right)\approx \vec{g},$$

where *a* is the total acceleration.

(4) $$\left[\begin{array}{c} a_{ave_{x}}\\ a_{ave_{y}}\\ a_{ave_{z}} \end{array}\right]=\left[\begin{array}{c} \mathrm{mean}\left(a_{x}\right)\\ \mathrm{mean}\left(a_{y}\right)\\ \mathrm{mean}\left(a_{z}\right) \end{array}\right]\approx \left[\begin{array}{c} g_{x}\\ g_{y}\\ g_{z} \end{array}\right]$$

$g_{x},g_{y},g_{z}$ are components of the gravity vector. According to Mizell, the acceleration along the vertical axis can be estimated as:

(5) $${\vec{a}}_{up}\approx \frac{\vec{a}\cdot {\vec{a}}_{ave}}{{\vec{a}}_{ave}\cdot {\vec{a}}_{ave}}{\vec{a}}_{ave},$$

Therefore, the acceleration along the horizontal plane can be estimated as:

(6) $${\vec{a}}_{h}=\vec{a}-{\vec{a}}_{up},$$

which is mainly applied to pedestrian activities and low-acceleration activities. For more general cases, leveled vertical and horizontal accelerations can be deduced from calculated pitch and roll during the strap down mechanization process. Analyzing along the forward and perpendicular axes is difficult because it requires the estimation of the heading, which is also difficult to calculate correctly. However, the magnitude of the leveled horizontal acceleration can be used as a variable to extract features [39]. In some previous studies, researchers used rotation matrix to transform magnetometer measurements to horizontal. In this study, we also transformed magnetometer measurements to horizontal first and then used magnetic field intensity proportion of each axis as the features. The following formulas present the detailed computation procedure:

(7) $${MagPercent}_{x/y/z}=\frac{{\left|Mag\right|}_{x/y/z}}{{\left|Mag\right|}_{x}+{\left|Mag\right|}_{y}+{\left|Mag\right|}_{z}}$$

where $Mag_{x},Mag_{y},Mag_{z}$ are processed magnetometer x−,y−,z− measurements.

#### 2.2.4. Body Acceleration Extraction

Accelerometer measurement is the sum of body acceleration and local gravity accelerations. We can use low-pass filter to obtain body acceleration from total acceleration. The procedure is presented in Algorithm 1.
**Algorithm 1** Body Acceleration Vector Extraction.**Input:**1. Low-pass filter coefficient α2. Total acceleration measurement vector ($TotalAcc_{x}$,$TotalAcc_{y}$,$TotalAcc_{z}$)3. Gravity vector ($Gravity_{x}$,$Gravity_{y}$,$Gravity_{z}$)**Output:** Body acceleration ($BodyAcc_{x}$,$BodyAcc_{y}$,$BodyAcc_{z}$)**If** Total acceleration measurement vector update$Gravity_{x}$ = α * $Gravity_{x}$ + (1−α) * $TotalAcc_{x}$$Gravity_{y}$ = α * $Gravity_{y}$ + (1−α) * $TotalAcc_{y}$$Gravity_{z}$ = α * $Gravity_{z}$ + (1−α) * $TotalAcc_{z}$$BodyAcc_{x}$ = $TotalAcc_{x}$ − $Gravity_{x}$$BodyAcc_{y}$ = $TotalAcc_{x}$ − $Gravity_{x}$$BodyAcc_{z}$ = $TotalAcc_{x}$ − $Gravity_{x}$

### 2.3. Feature Extraction

In this study, we obtained measurements from MEMS sensors, such as accelerometer, gyroscope, magnetometer, and light. However, raw measurement series were insufficient to construct features. We calculated some characteristics to construct an efficient feature vector for improving recognition capability. Table 1 summarizes the definitions of these commonly used features.

### 2.4. Traditional ML Classification Methods

#### 2.4.1. k-Nearest Neighbor

kNN algorithm is a supervised ML algorithm that can be used for both classification and regression predictive problems. However, it is mainly used for classification predictive problems in industry. kNN is a non-parametric and lazy learning algorithm. Non-parametric means no assumption is imposed for underlying data distribution. In other words, the model structure is determined from the dataset. This is helpful in practice as most real-world datasets do not follow mathematical theoretical assumptions. Lazy algorithm means it does not need any training data points for model generation. All training data are used in the testing phase. Thus, the training phase is fast and the testing phase is slow and costly. Costly testing phase consumes time and memory. In the worst case, kNN needs much time to scan all data points, and scanning all data points will require much memory for storing training data [40].

#### 2.4.2. Random Forrest

RF and random decision forests are ensemble learning methods for classification, regression, and other tasks that operate by constructing a multitude of DT at training time and outputting the class that is the mode of the classes (classification) or mean prediction (regression) of the individual trees. Random decision forests correct for the DT habit of over-fitting to their training set [41].

#### 2.4.3. Support Vector Machine

In ML, SVM are supervised learning models with associated learning algorithms that analyze data used for classification and regression analysis. Given a set of training examples, each marked as belonging to one or the other of two categories, an SVM training algorithm builds a model that assigns new examples to one category or the other. Thus, this method a non-probabilistic binary linear classifier. An SVM model is a representation of the examples as points in space and is mapped to ensure that the examples of the separate categories are divided by a clear gap that is as wide as possible. New examples are then mapped into that same space and predicted to belong to a category based on the side of the gap on which they fall.

#### 2.4.4. Decision Tree

DT is a non-parametric supervised learning method used for classification and regression. The goal is to create a model that predicts the value of a target variable by learning simple decision rules inferred from the data features. DT is commonly used in operations research, specifically in decision analysis, to help identify a strategy that likely reaches the goal. They are also a popular tool in ML.

#### 2.4.5. Naive Bayes

Naive Bayes is a simple technique for constructing classifiers. These models that assign class labels to problem instances, which are represented as vectors of feature values. The class labels are drawn from some finite set. No single algorithm can be used to train such classifiers, but a family of algorithms based on a common principle can be utilized. All naive Bayes classifiers assume that the value of a particular feature is independent of the value of any other features, given the class variable. Naive Bayes only requires a small number of training data to estimate the parameters necessary for classification. A naive Bayes classifier is a probabilistic ML model that is used for classification tasks [40].

#### 2.4.6. Neural Network

**Figure 2:**
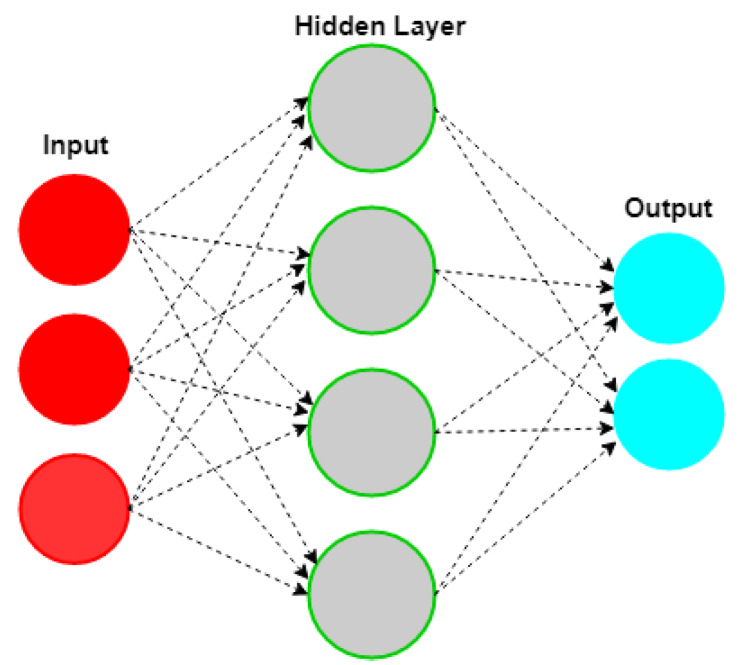
Structure of ANN.

An ANN is an interconnected group of nodes and is inspired by a simplification of neurons in a brain. Here, each circular node represents an artificial neuron and an arrow represents a connection from the output of one artificial neuron to the input of another. Neural networks are multi-layer networks of neurons that we use to classify things and make predictions. Figure 2 is the diagram of a simple NN with three inputs, two outputs, and one hidden layer of neurons.

#### 2.4.7. Stochastic Gradient Descent

SGD (Stochastic Gradient Descent) is an iterative method for optimizing an objective function with suitable smoothness properties. It is called stochastic because the method uses randomly selected samples to evaluate the gradients. Thus, SGD can be regarded as a stochastic approximation of gradient descent optimization. SGD has been successfully applied to large-scale and sparse machine learning problems often encountered in text classification and natural language processing. SGD is efficient and can be easily implemented. However, SGD requires several hyperparameters, such as the regularization parameter and the number of iterations. SGD is also sensitive to feature scaling.

### 2.5. Deep Learning Methods of Classification

In this study, we constructed deep learning models by LSTM network and CNN. Training was performed on a Win64 personal computer (Intel-R Core-TM i5-8250U CPU @1.6GHz). Tensorflow was the deep learning framework we used. LSTM network and CNN are introduced in detail below.

#### 2.5.1. LSTM Network

LSTM is an improved RNN (Recurrent Neural Network) deep learning model. Notably, RNN has problems with gradient vanishing or explosion. LSTM is a complicated function that learns to control the flow of information, to prevent the vanishing gradient and to allow the recurrent layer to easily capture long-term dependencies. LSTM is also explicitly designed to avoid the long-term dependency problem. LSTM was introduced by Hochreiter and Schmidhuber (1997) and was refined and popularized in several further studies. LSTM works tremendously well on the various problem and is now widely used [42].

**Figure 3:**
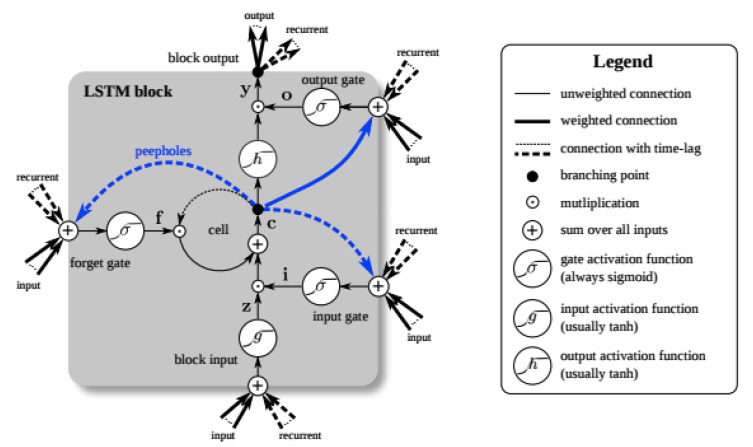
Training and test situation by raw dataset.

LSTM is used in the field of deep learning. Unlike standard feed-forward neural networks, LSTM has feedback connections. It processes not only single data points (e.g., images) but also entire sequences of data (e.g., speech or video). A common LSTM unit is composed of a cell, an input gate, an output gate, and a forget gate. The cell remembers values over arbitrary time intervals, and the three gates regulate the flow of information into and out of the cell. LSTM networks are suitable for classifying, processing, and making predictions based on time series data because lags of unknown duration may exist between important events in a time series. The theoretical architecture of all hyper-parameters of the LSTM network is presented in Figure 3.

#### 2.5.2. CNN Network

In deep learning, CNN is a class of deep neural networks. Its name indicates that the network uses a mathematical operation called convolution. Convolution is a specialized kind of linear operation. Convolutional networks are simply neural networks that use convolution in place of general matrix multiplication in at least one of their layers. A CNN consists of an input and an output layer, and multiple hidden layers [43]. The hidden layers of a CNN typically consist of a series of convolutional layers that convolve with multiplication or other dot product. The activation function is commonly a RELU layer and is subsequently followed by additional convolutions, such as pooling layers, fully connected layers, and normalization layers. They are referred to as hidden layers because their inputs and outputs are masked by the activation function and final convolution. The final convolution, in turn, often involves back-propagation to accurately weight the end product.

### 2.6. Real-Time Recognition Using Trained Model

#### Transformation of Trained Model

**Figure 4:**
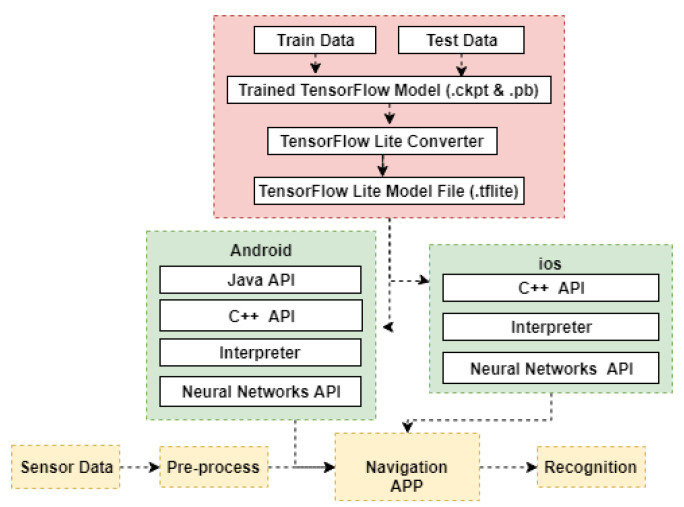
Filtered MEMS measurements.

Figure 4 shows the workflow of how we used a trained model file in a mobile device. In this study, we used Windows version Tensorflow to train measurements for obtaining a .pb model. The model precision of the model was validated using the test data. Then, a .pb file was converted to .tflite file, which is a lightweight model file that can be run on mobile devices. Green blocks show the mechanism of Android and iOS using a .tflite model file. Yellow blocks show how our Android application uses sensor data and .tflite model file to recognize pedestrian motion mode or posture in real-time.

### 2.7. Analysis and Evaluation of Recognition Results

When a model is trained, its accuracy must be validated using test data; a confusion matrix is often used to evaluate prediction precision. Table 2 presents a four-class classifier example.

$n_{ij}$ indicates the prediction is j−class, but the true label is i−class. If i=j, then the prediction is right. On basis of the above-mentioned confusion matrix, several statistics are proposed to evaluate prediction model precision. Table 3 lists the detailed information of these statistics for prediction evaluation.

Four commonly used statistics to evaluate recognition situation and training model, namely ‘accuracy’, ‘recall’, ‘f-measure’, and ‘precision’, were used in this study. Classification accuracy is the proportion of correctly classified examples. F-1 is a weighted harmonic mean of precision. Precision is the proportion of true positives amongst instances classified as positive. Recall is the proportion of true positives amongst all positive instances in the data.

### 2.8. Navigation Location Update

The core target of motion mode or posture recognition is to optimize navigation algorithm and improve navigation location precision. Table 4 presents the pedestrian navigation update strategies of different motion modes [45].

Table 4 shows that different navigation strategies should be used for different motion modes. For example, horizontal location should not be updated in elevator. Thus, we need to know if the pedestrian is in the stage of elevator.

## 3. Validation and Experiment

### 3.1. Pedestrian Motion Mode Recognition

#### 3.1.1. Test Description and Preprocessing

**Figure 5:**
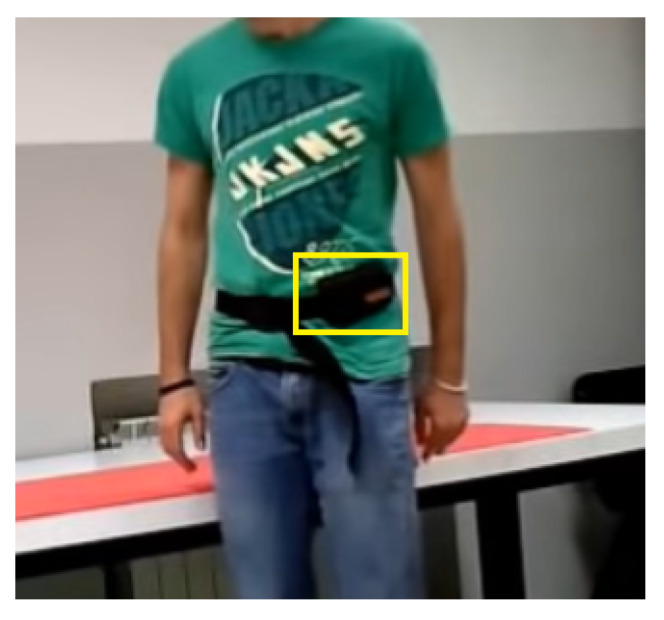
Pedestrian taking the test with smartphone on waist.

We used the public dataset that can be found on UCI website [46]. The experiments were performed with a group of 30 volunteers within the age range of 19–48 years. Each person performed six activities (i.e., Walking, Upstairs, Downstairs, Sitting, Standing, and Laying) while wearing a smartphone (Samsung Galaxy S II) on the waist, like the posture in Figure 5.

**Figure 6:**
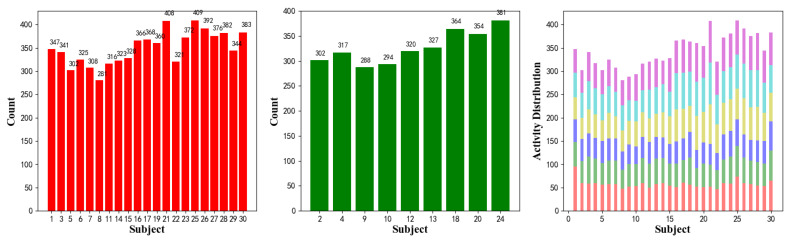
Human activity recognition dataset of UCI. **Left**: activity distributions of training data and related subjects; **Middle**: dataset distribution of validation data; **Right**: six activities’ distribution of 30 subjects.

By using the smartphone’s embedded accelerometer and gyroscope, the testers captured three axial linear accelerations and three axial angular velocities at a constant rate of 50 Hz. The obtained dataset was randomly partitioned into two sets, where 70% of the volunteers were selected for generating the training data and 30% were for the test data. The sensor signals (accelerometer and gyroscope) were preprocessed by applying noise filters and then sampled in fixed-width sliding windows of 2.56 s and 50% overlap (128 readings/window). The sensor acceleration signal, which had gravitational and body motion components, was separated using a Butterworth low-pass filter into body acceleration and gravity. The gravitational force was assumed to have only low-frequency components. Therefore, a filter with a 0.3 Hz cutoff frequency was used. From each window, a vector of features was obtained by calculating variables from the time and frequency domain. The detailed information is listed in Figure 6 and Table 5.

The left graph in Figure 6 presents the activity distributions of training data and related subjects; the middle graph shows the dataset distribution of validation data; and the right graph shows six activities’ distribution of 30 subjects.

The total number of all labels is 10,299, and they belong to six activities.

#### 3.1.2. Classification Using Traditional ML Methods

In past years, several ML algorithms had been developed to solve classification. In this study, traditional methods, namely SGD, Naive Bayes, DT, kNN, RF, NN, and SVM, were used to classify the test data. Table 6 shows the classification results.

#### 3.1.3. Classification Using Deep Learning

Apart from the seven ML classification methods, we also developed two deep learning models: one was based on LSTM network, and the other one was designed using CNN.

**Figure 7:**
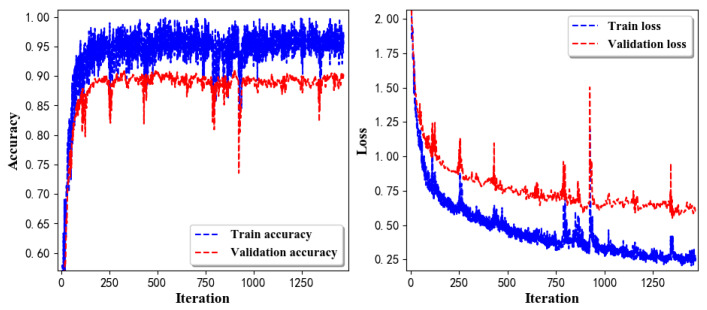
Accuracy (**left**) and loss (**right**) information of training and validation procedure.

**Figure 8:**
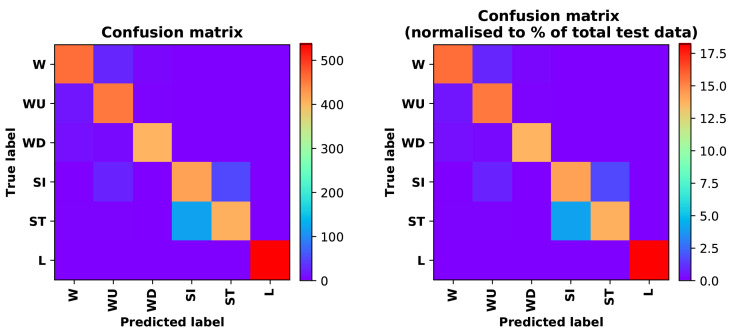
**left** graph is the confusion matrix of classification test using the model trained by LSTM network; **right** is the normalised confusion matrix.

**Figure 9:**
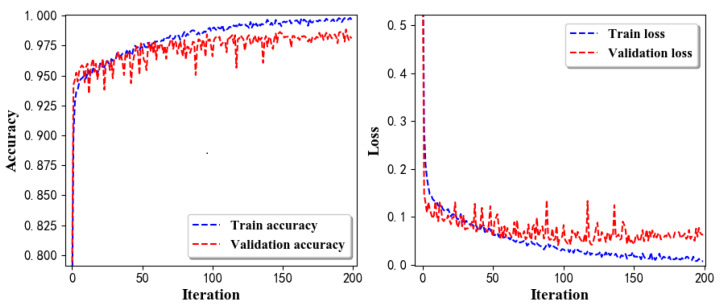
Accuracy (**left**) and loss (**right**) information of training and validation procedure.

**Figure 10:**
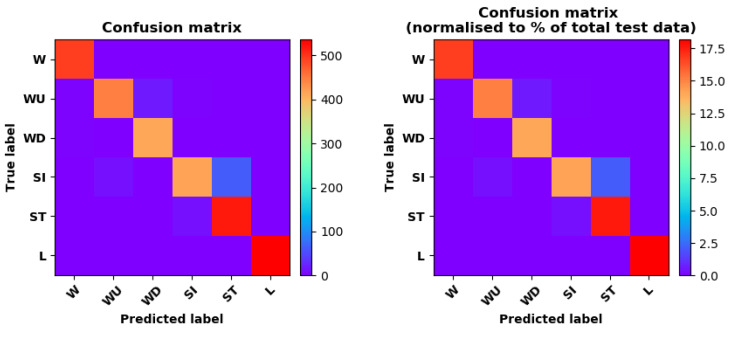
**left** graph is the confusion matrix of classification test using the model trained by CNN network; **right** is the normalised confusion matrix.

1. Training Recognition Model Using LSTM Network
  Table 7 presents the structure of the designed LSTM network model. Notably, three LSTM layers are configured. In the table, ‘None’ denotes the batch size. We set the batch size to 200 and adopted the ‘adam’ optimizer in this test. These parameters in the table were only used for motion model recognition. The network structure in the next section is the same, but the parameters are updated.
  Figure 7 presents the accuracy and loss information after training the data using the LSTM model: training accuracy exceeds 0.95, the validation precision exceeds 0.9, and the loss is close to 0.6.
  Figure 8 presents the confusion matrix graph. The true labels were obtained from the test data, and the predictions were the recognition results obtained from above trained model using the test data.
2. Training Recognition Model Using CNN
  Our CNN architecture involves four consecutive blocks, each including a convolutional and RELU activation layer. Each convolutional kernel performs a 2D convolution over the time dimension, for each sensor channel independently. Preliminary experiments show that models with convolutions performed across all sensor channels degrade performances on the opportunity dataset. Similar to MLP (Multi-Layer Perceptron), adding a batch normalization layer right after the input layer yields significant performance improvements.
  Table 8 presents the structure of the designed CNN model that contains four 2D CNN layers. In the table, ‘None’ denotes the batch size. We set the batch size to 200 and adopted the ‘adam’ optimizer in this test. These parameters list in the table were only used for motion model recognition. The network structure in the next section is the same, but the parameters are updated.
  Figure 9 presents the training accuracy and loss graphs. The blue curve represents the training accuracy and loss, and the red curve denotes the validation precision and loss. The training and validation accuracies exceed 0.95, and the losses converged below 0.1.
  Figure 10 presents the confusion matrix graph. The true labels were obtained from the test data, and the predictions were recognized from the trained model using the test data.

### 3.2. Smartphone Posture Recognition

#### 3.2.1. Test Description and Preprocessing

**Figure 11:**
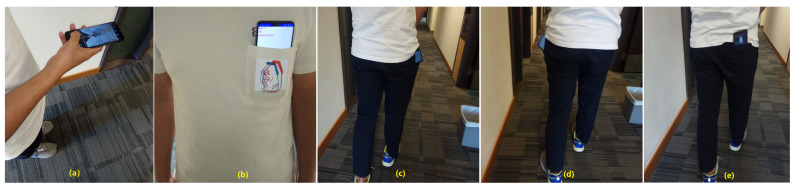
Smartphone posture recognition experiment. (**a**) is holding smartphone; (**b**) is in the chest pocket; (**c**) is in the right trouser pocket; (**d**) is in the left trouser pocket; (**e**) is in the buttock pocket

In this experiment, we designed nine test postures. Figure 11a shows a pedestrian holding a smartphone horizontally. Figure 11b presents a pedestrian with a smartphone in the chest pocket while keeping the smartphone screen forward; the backward posture is also designed. Figure 11c,d shows a pedestrian with a smartphone in the right or left trouser pocket while keeping the smartphone screen inward or outward. Figure 11e presents a pedestrian with the test phone in the buttock pocket while keeping the smartphone screen forward or backward. In the following sections, we use ‘H’ to denote the horizontal posture for the case in Figure 11a. We use ‘CB’ and ‘CF’ to denote backward and forward postures for the case in Figure 11b. ‘RLB’ and ‘RLF’ are used to denote backward and forward postures for the case in Figure 11c. ‘LLB’ and ‘LLF’ denote backward and forward postures for the case in Figure 11d. ‘ARB’ and ‘ARF’ express backward and forward postures for the case in Figure 11e. We executed nine separate experiments; each test collected 26,000 records. On the basis of these data, we selected 20,000 items for training and 6000 records for evaluation. In total, 180,000 groups of records were used for training, and 54,000 records were used for evaluation.

We collected accelerometer, gyroscope, magnetometer, and light measurements from MEMS sensors. We used the above-mentioned preprocessing methods to process these data and filtered the noise of raw measurements.

**Figure 12:**
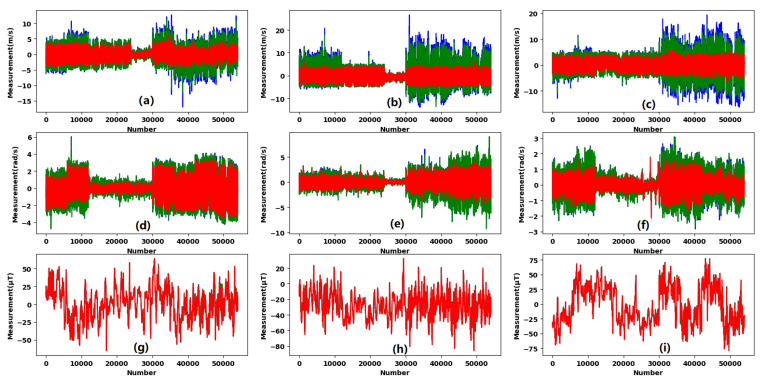
MEMS measurement processing strategies; (**a**)–(**c**) are filtered accelerometer *x*-, *y*-, *z*-axis measurements; (**d**)–(**f**) are filtered gyroscope *x*-, *y*-, *z*-axis measurements; (**g**)–(**i**) are filtered magnetometer *x*-, *y*-, *z*-axis measurements.

The blue curve in Figure 12 represents the raw measurements obtained from MEMS sensors. The green curve is the low-pass filter result, and the red curve is the end filter result based on the aforementioned preprocessing methods. Figure 12a–c shows the filtering results of accelerometer *x*-, *y*-, *z*-axis measurements, respectively. Figure 12d–f shows the filtering results of gyroscope *x*-, *y*-, *z*-axis measurements, respectively. Figure 12g–i shows the filtering results of magnetometer *x*-, *y*-, *z*-axis measurements, respectively.

#### 3.2.2. Classification Using Traditional ML Methods

We designed multiple feature strategies based on accelerometer, gyroscope, magnetometer, and light measurements. On the basis of these strategies, we used different ML classification methods to train and test these data. The following section presents five validation results.

Raw feature vector is [AccX, AccY, AccZ, GyroX, GyroY, GyroZ, MagX, MagY, MagZ]. [AccX, AccY, AccZ] are the accelerometer *x*-, *y*-, *z*-axis measurements. [GyroX, GyroY, GyroZ] are the gyroscope *x*-, *y*-, *z*-axis measurements. [MagX, MagY, MagZ] are the magnetometer *x*-, *y*-, *z*-axis measurements. The same meanings are applied in the following sections.

Table 9 shows that SGD has the lowest classification precision with only 0.080. NN obtains the highest classification precision, but the value is still only 0.478, which is lower than 0.5. Thus, we add Light measurement as one feature. The feature vector is [AccX, AccY, AccZ, GyroX, GyroY, GyroZ, MagX, MagY, MagZ, Light],where Light is the light sensor output measurement.

As shown in Table 10, the highest classification precision is only 0.465, which is still less than 0.5. Thus, we processed the raw measurements based on the aforementioned strategies to obtain efficient features. The feature vector is [FilterBodyAccX, FilterBodyAccY, FilterBodyAccZ, FilterGyroX, FilterGyroY, FilterGyroZ, MagPercentX, MagPercentY, MagPercentZ, Light]. Total acceleration was replaced by body acceleration. FilterBodyAccX, FilterBodyAccY, and FilterBodyAccZ are the filtered body accelerations, and body acceleration was computed using Algorithm 1. FilterGyroX, FilterGyroY, and FilterGyroZ are the filtered gyroscope measurements. MagPercentX, MagPercentY, and MagPercentZ were derived from magnetometer measurements based on Equation (7).

Table 11 show the validation results. Evidently, precision is improved, and the highest is up to 0.747. Therefore, processed features improves recognition precision. We also removed light measurement from features to verify if light was useful for the classification. The updated feature vector is [FilterBodyAccX, FilterBodyAccY, FilterBodyAccZ, FilterGyroX, FilterGyroY, FilterGyroZ, MagPercentX, MagPercentY, MagPercentZ].

Table 12 presents the classification results. Notably, kNN has the highest precision but has only little improvement compared with the above-mentioned experiment. We designed the feature vector as [FilterBodyAccX, FilterBodyAccY, FilterBodyAccZ, FilterGyroX, FilterGyroY, FilterGyroZ]. Table 13 presents the results.

As shown in Table 13, NN has the highest precision. The precision values of RF and NN exceed 0.8. Thus, we think these features are more efficient for classification than the others.

#### 3.2.3. Classification Using Deep Learning

**Figure 13:**
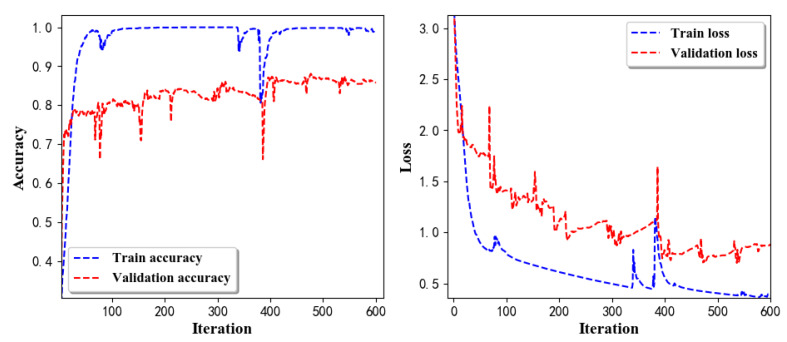
Accuracy (**left**) and loss (**right**) information of training and validation procedure.

**Figure 14:**
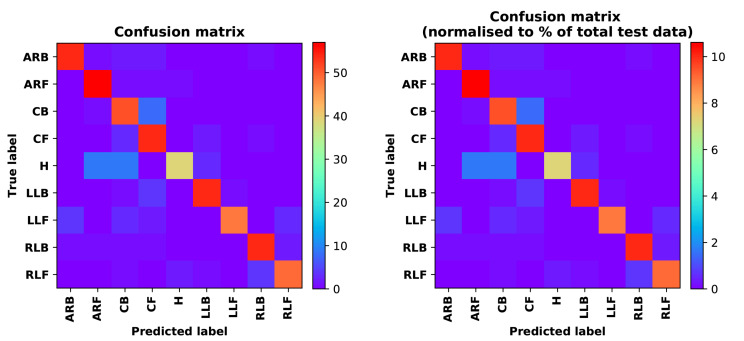
**left** graph is the confusion matrix of classification test using the model trained by LSTM network; **right** is the normalised confusion matrix.

**Figure 15:**
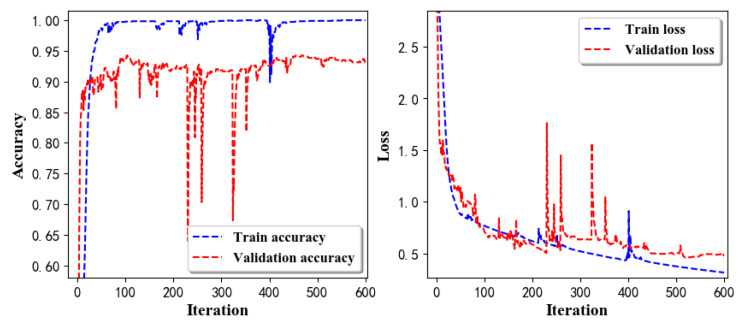
Accuracy (**left**) and loss (**right**) of training and validation procedure using filtered measurements.

**Figure 16:**
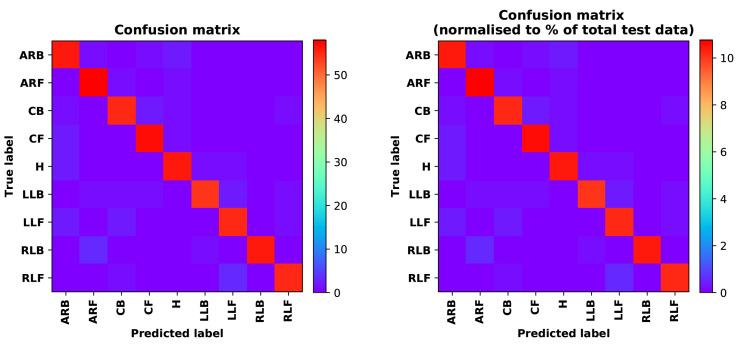
**left** graph is the confusion matrix of classification test using the model trained by LSTM network with filtered measurements; **right** is the normalised confusion matrix.

**Figure 17:**
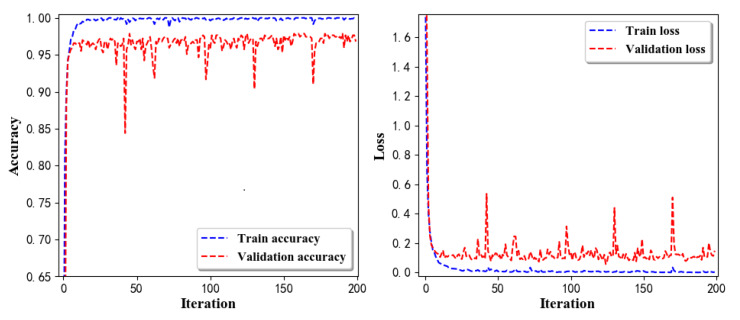
Accuracy (**left**) and loss (**right**) information of training and validation procedure.

**Figure 18:**
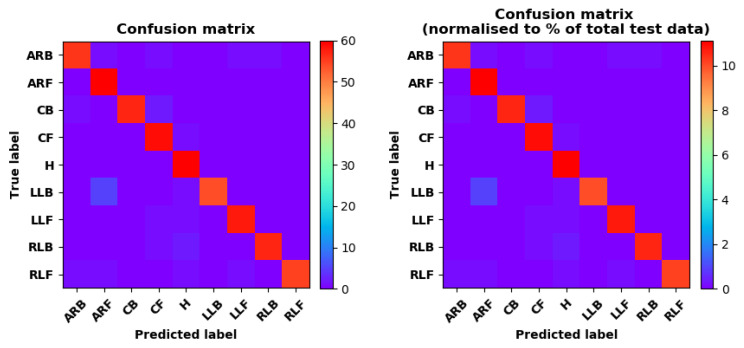
**left** graph is the confusion matrix of classification test using the model trained by CNN network; **right** is the normalised confusion matrix.

1. Training Recognition Model Using LSTM Network
  We used the designed LSTM model to train the raw data. Figure 13 presents the accuracy and loss information in the process of training and validation.
  Figure 14 presents two confusion matrix graphs; the right one is the normalized confusion matrix. The confusion matrix shows large prediction errors. Large proportions of ‘CB’ were recognized as ‘CF’. The same situation occurs for ‘H’. Moreover, most predictions were recognized as ‘ARF’ and ‘CB’.
  Figure 14 presents the confusion matrix graph. The true labels were obtained from the test data, and the predictions were recognized from the trained model using the test data. From the results in Figure 14, we still find some bright spots; they are in the off diagonals, meaning the wrong classification numbers or proportion. In the above graph, for example, several “CB” were recognized as “CF”, and many “H” were classified as “ARF” and “CB”. Thus, we know the training model still has some drawbacks and needs improvement. We also used the above designed LSTM model to train the filtered data. Figure 15 presents accuracy and loss information of training and validation data.
  Figure 16 presents the confusion matrix graph. The true labels were derived from the test data, and predictions were recognized from the trained model using the test data. The confusion matrix graph below shows that correct prediction takes up the highest proportion. Most predictions of the nine postures are correct.
2. Training Recognition Model Using CNN
  Apart from the aforementioned LSTM methods, we also trained the preprocessed data using CNN. The CNN structure is same, but the parameters are updated here. Figure 17 presents the accuracy and loss of the training and validation results.
  Figure 18 shows the confusion matrix of prediction. The model was trained by CNN with the preprocessed measurements. The confusion matrix graph below indicates that the correct prediction takes up the highest proportion. Most predictions of the nine postures are correct. Overall, no large difference from the LSTM network trained results is observed.
  Figure 18 presents the confusion matrix graph. The true labels were obtained from the test data, and the predictions were recognized from the trained model using the test data.

### 3.3. Real-time Activity Recognition Test Combining Pedestrian Motion and Smartphone Posture

#### 3.3.1. Test Description and Preprocessing

**Figure 19:**
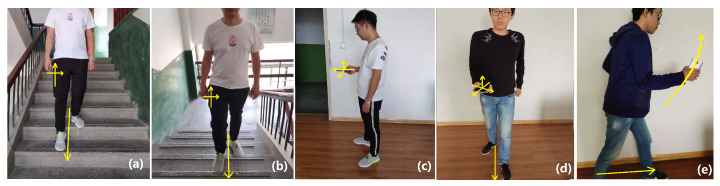
Real-time recognition test of comprehensive pedestrian activities.(**a**) is downstairs; (**b**) is upstairs; (**c**) is standing; (**d**) is walking; (**e**) is jogging.

In the above two experiments, motion modes were verified with a fixed posture smartphone placed in the waist pocket. In the smartphone posture recognition experiment, the pedestrian walked on the same level ground and switched the smartphone to different postures. In actual situation, the pedestrian navigation procedure combines motion mode and smartphone posture together and cannot separate them. Thus, in the real-time recognition experiment, we designed five commonly encountered comprehensive activities based on real pedestrian navigation situations. Figure 19a presents a tester who is walking downstairs with a smartphone placed in the right leg trousers pocket. The smartphone screen is kept inward. In Figure 19b, a tester is walking upstairs with a smartphone placed in the pocket. The posture is similar to that in Figure 19a, Figure 19c presents a pedestrian who is standing on the ground with a smartphone in hand while keeping the smartphone horizontally. Figure 19d shows a pedestrian with a smartphone in hand and who is walking forward. The smartphone screen is kept upward. Figure 19e presents our jogging situation, in which a pedestrian holds a smartphone in the right hand while swinging the arm and the smartphone. Our real-time recognition tests were performed on 1 October 2019, in the 4# building of Chang’an University. In our tests, the update frequency of MEMS sensors was 100 Hz. Thus, we set the window size to 200 to collect 2 s measurements.

Each experiment exceeded 10 min and recorded accelerometer, gyroscope, magnetometer, and light measurements from MEMS sensors based on Android API. We collected 77,400 records of ‘Downstairs’, 75,000 records of ‘Upstairs’, 103,600 records of ‘Standing’, 73,000 records of ‘Walking’, and 72,600 records of ‘Jogging’ for training.

#### 3.3.2. Real-time Recognition of Comprehensive Pedestrian Activities

On the basis of the collected measurements of comprehensive pedestrian activity experiment, we decided to train a recognition model using CNN. Several operators of LSTM network still cannot be run in mobile side to date. Thus, we used CNN to train the model. Different processing strategies are summarized as follows.

**Figure 20:**
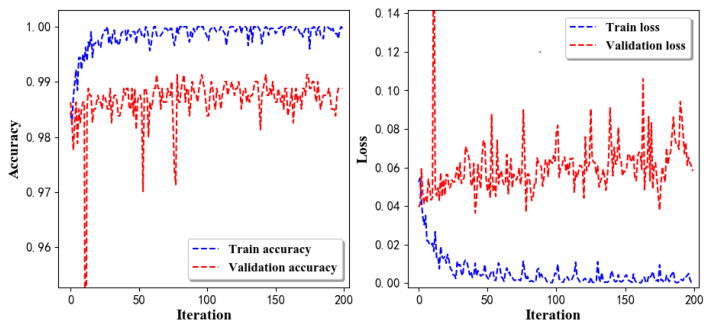
Accuracy (**left**) and loss (**right**) information of training and validation procedure.

**Figure 21:**
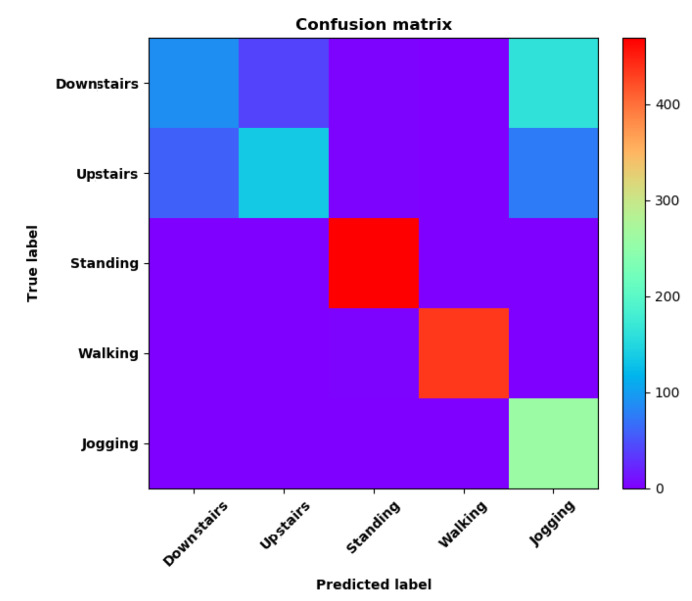
Confusion matrix of classification test using the model trained by CNN network.

**Figure 22:**
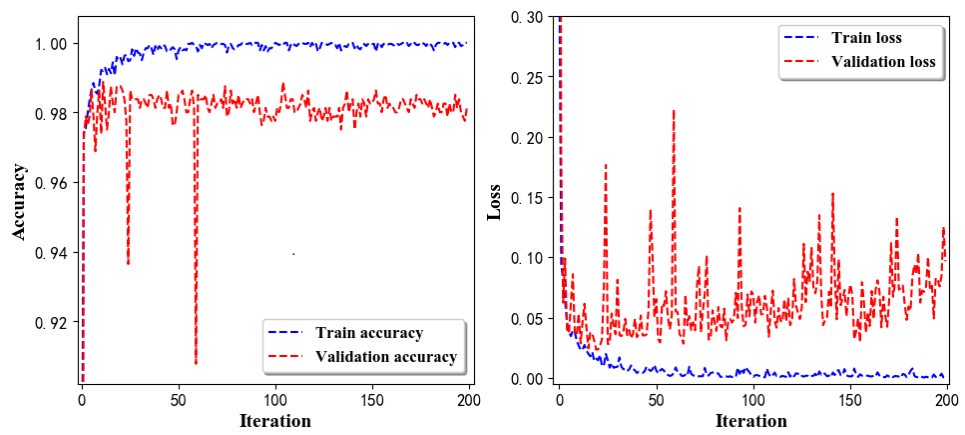
Accuracy (**left**) and loss (**right**) information of training and validation procedure with light measurements.

**Figure 23:**
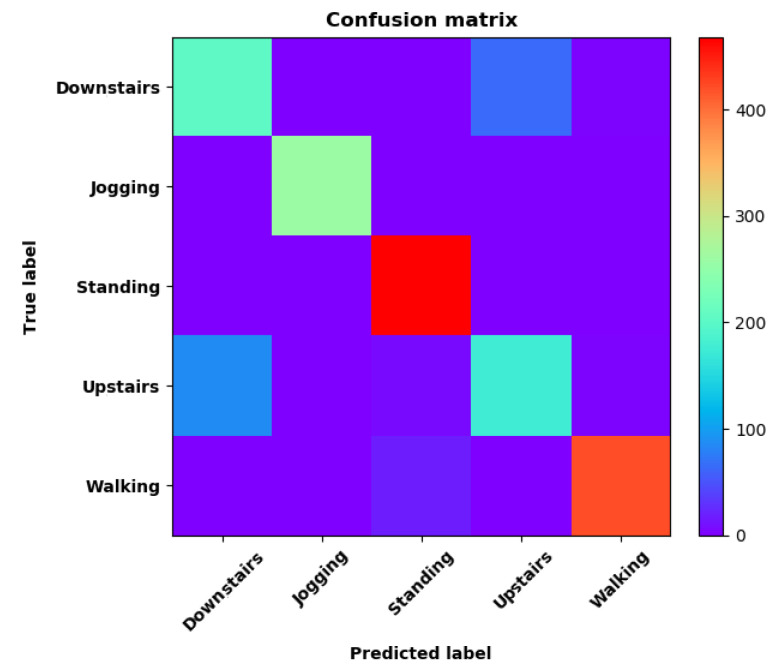
Confusion matrix of classification test using the model trained by CNN network with light measurements.

1. Training Recognition Model using Gyroscope + Accelerometer + Magnetometer Measurements
  We trained the data and used the feature vector [FilterBodyAccX, FilterBodyAccY, FilterBodyAccZ,FilterAccX, FilterAccY, FilterAccZ, FilterGyroX, FilterGyroY, FilterGyroZ, MagPercentX, MagPercentY, MagPercentZ] with 12 features under the window size of 200. Figure 20 presents the accuracy and loss graphs of training procedure.
  Validation tests were performed in the same test fields, and five separate tests were done. Figure 21 shows the confusion matrix of real-time test.
  Figure 21 presents the confusion matrix graph. The true labels were derived from the test data; predictions were recognized from the above-trained model using the test data. The confusion matrix indicates that ‘Downstairs’ and ‘Upstairs’ have large prediction errors. Among the 296 instances of ‘Downstairs’, the number of correct predictions was only 89; 42 were recognized as ‘Upstairs’ and 162 were detected as ‘Jogging’. Therefore, the accuracy is only 30.07%. With regard to ‘Upstairs’, the number of correct predictions is 136; 76 of these instances ertr recognized as ‘Jogging’ and 59 were detected as ‘Downstairs’. Therefore, the accuracy was only 49.64%. ‘Standing’, ‘Walking’, and ‘Jogging’ had high correct prediction proportions. The total prediction accuracy is 79.87%.
2. Training Recognition Model Using Gyroscope + Accelerometer + Magnetometer + Light Measurements
  In the above section, we found that ‘Downstairs’ and ‘Upstairs’ had high incorrect recognition rate. Thus, we added ‘Light’ as one of the features. The feature vector was [FilterBodyAccX, FilterBodyAccY, FilterBodyAccZ,FilterAccX, FilterAccY, FilterAccZ, FilterGyroX, FilterGyroY, FilterGyroZ, MagPercentX, MagPercentY, MagPercentZ, Light] with 13 features under the window size of 200. Figure 22 presents the accuracy and loss graphs of training and validation procedure.
  Test was performed in the same test fields, and five separate experiments were done. Figure 23 presents the confusion matrix graph.
  Figure 23 presents the confusion matrix graph; true labels were obtained from the test data and the predictions were recognized from the above trained model using the test data. Among the 270 instances of ‘Downstairs’, the correct prediction rate was 75.19%; 65 of these instances were recognized as ‘Upstairs’. Among the 274 instances of ‘Upstairs’, 177 were predicted correctly; 87 of these instances were detected as ‘Downstairs’. Therefore, the correct prediction rate was only 64.60%.

### 3.4. Pedestrian Navigation Test

#### 3.4.1. Test Description

**Figure 24:**
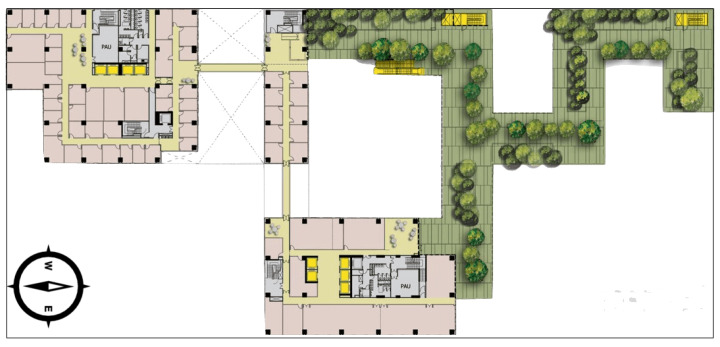
The plan of test field.

A pedestrian navigation experiment was performed in Block Z of Hong Kong Polytechnic University; the test field was located on the sixth floor of the building. Figure 24 presents the test site environment.

On the south side, there are tall buildings, and on the north, near the buildings, there is an open-sky platform. Our experiment was mainly performed on the platform, where the pedestrian walked from indoors to outside. In the test procedure, the pedestrian held a smartphone in hand, and mixed ‘Walking’, ‘Standing’, and ‘Jogging’ activities together. The experiment was executed using a Huawei Mate20 Android phone, on which our navigation application was deployed; the details of navigation scheme were introduced in our previous study [1]. The recognition model trained in Section 3.3 was applied in this experiment.

#### 3.4.2. Navigation Test Result

**Figure 25:**
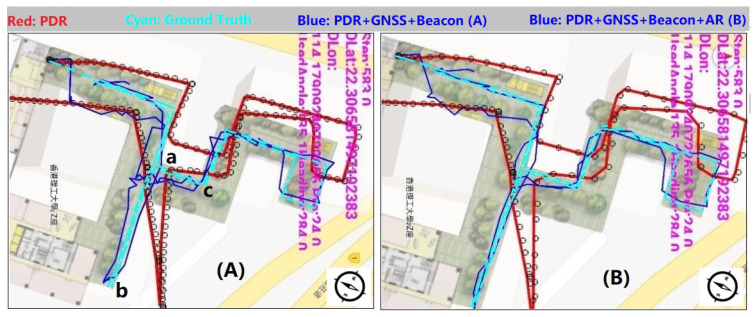
Graph (**A**) is the navigation test result without AR(Activity Recognition); (**B**) uses AR.

In the experiment, three navigation strategies were employed: ‘PDR’, ‘PDR + GNSS + Beacon’ fusion positioning, and ‘PDR + GNSS + Beacon + Activity Recognition’. Figure 25 presents these strategies’ positioning results.

In Figure 25, the red line and the cyan line are pure PDR results and the pedestrian walking route separately. The blue line, in Figure 25A, denotes ‘PDR + GNSS + Beacon’ fusion result; in Figure 25B, it denotes ‘PDR + GNSS + Beacon + Activity Recognition’ location results. ‘a’, ‘b’ and ‘c’ are corner points; the tester walked along ‘c-a-b’, mixing ‘walking’, ‘standing’ and ‘jogging’ activities together.

## 4. Discussion

We designed four main experiments to verify recognition performance using smartphone MEMS sensors’ measurements. We used traditional mainstream ML classification methods to recognize pedestrian motion modes and smartphone postures. We also trained recognition models based on the designed deep learning models constructed by LSTM network and CNN. Based on the actual requirements of pedestrian navigation, we designed five comprehensive postures and developed an Android application to recognize these postures in real-time. The following subsections discuss these experimental results in detail.

### 4.1. Motion Mode Recognition

In the motion mode classification experiment, motion modes were verified using seven ML classification methods and two deep learning methods, designed by LSTM network and CNN.

From the test results of seven traditional ML classification methods mentioned in Table 6, we find SVM has the highest precision with a value of 0.899 (89.90%). Table 14 and Table 15 list the test results that are evaluated by the proposed LSTM and CNN.

The accuracies are up to 90.74% and 90.74% in Table 14 and Table 15, respectively. Both are higher than the SVM classification accuracy of 89.90%.

From the results above, we can conclude that the two deep learning methods are useful in recognizing the motion modes. The classification accuracy of our designed LSTM and CNN models are also efficient. LSTM and CNN models have higher accuracy and precision than traditional ML classification methods.

### 4.2. Smartphone Posture Recognition

The second experiment was designed to recognize smartphone postures. We designed nine postures in this experiment. In smartphone posture classification experiment, smartphone postures were verified using seven ML classification methods and two deep learning methods, designed by LSTM network and CNN.

The test results of seven traditional ML classification methods mentioned in Table 9, Table 10, Table 11, Table 12 and Table 13 show that, when raw accelerometer, gyroscope, and magnetometer measurements were used, NN obtained the highest accuracy with a value of 0.478 (47.8%). If light measurements were added, then neural network also obtained the highest accuracy with a value of 0.465 (46.5%). If processed accelerometer, gyroscope, magnetometer and light measurements were used, then NN still obtained the highest accuracy with a value of 0.747 (74.7%). If only processed accelerometer, gyroscope, and magnetometer measurements were utilized, then kNN obtained the highest accuracy with a value of 0.757 (75.7%). We also tried to use processed accelerometer and gyroscope measurements without magnetometer and light. The results showed that NN had the highest precision with a value of 0.816 (81.60%).

Table 16 lists the evaluation results for the recognition model that was trained on the basis of the deep learning model constructed by LSTM networks and used raw measurements to create features. The classification accuracy was up to 85.77%. We also processed raw measurements and then trained the data using LSTM network model.

Table 17 lists the evaluation results for the model that was trained on the basis of LSTM network model and used filtered measurements as features. In Table 16 and Table 17, we can find that the accuracy is improved evidently. Therefore, our preprocessing algorithm is useful, and the designed LSTM network model is efficient.

Table 18 lists the evaluation results for the model that was trained on the basis of CNN model and used filtered measurements as features. Table 17 shows that the accuracy trained by CNN model is better than that by LSTM network model. The accuracy of the two deep learning methods is improved significantly compared with those of the seven machine learning methods. Therefore, the designed LSTM and CNN are useful.

### 4.3. Real-time Recognition of Comprehensive Pedestrian Activities

In this study, we aimed to train MEMS measurements to obtain a useful model to recognize pedestrian postures in real time. On the basis of previous experiments, we designed five comprehensive postures that combined frequently used motion modes and smartphone postures. In the real-time experiment, we collected accelerometer, gyroscope, magnetometer, and light measurements. In the first test, we trained the recognition model by using only accelerometer, gyroscope, and magnetometer measurements. Table 19 presents the statistical results.

The confusion matrix in Figure 21 shows that ‘Walking’, ‘Sitting’, and ‘Standing’ have high recognition rates. However, the model cannot classify ‘Upstairs’ and ‘Downstairs’. Most instances of the two activities were recognized to belong to others, and the two activities had high incorrect recognition rates. On the basis of previous studies, we added light measurement as a feature and trained a new recognition model using CNN model. Table 20 shows the evaluation results.

Table 19 and Table 20 show that the recognition accuracy is improved from 79.82% to 89.39%, and the improvement is significant. Therefore, light measurement is efficient to differentiate ‘Upstairs’ and ‘Downstairs’ from other activities and improves recognition accuracy. Overall, real-time recognition accuracy is up to 89.39%. Therefore, the solution is useful to recognize pedestrian activities and vital to update related algorithms during navigation.

### 4.4. Navigation Test Result Analysis

In pedestrian navigation experiment, ‘walking’, ‘standing’ and ‘jogging’ activities were mixed together. We processed the measurements using the three strategies, ‘PDR’, ‘PDR + GNSS + Beacon’, and ‘PDR + GNSS + Beacon + Activity Recognition’. Figure 25 presents the three schemes’ processing results. We find pure PDR (red line) has the largest bias compared with the true route (cyan line); obviously, the fusion result has big improvement and the navigation locations are close to the pedestrian moving route. In this experiment, we aimed to verify if activity recognition is useful to improve positioning precision, thus we processed the same data with the two schemes ‘PDR + GNSS + Beacon’ and ‘PDR + GNSS + Beacon + Activity Recognition’. In the procedure of fusion, once activities were recognized, the program would switch to different processing strategies; for example, when the pedestrian was ‘standing’, the location could not be updated; and, when ‘jogging’ was found, the program would update GNSS weight automatically in the process of fusion, to reduce the impact of swinging. Overall, the blue line in Figure 25B is much closer to the pedestrian moving route; from our statistic, in the route ‘c-a-b’ the mean bias reduces more than 1.1 m compared with the result in Figure 25A, which means it has higher precision.

## 5. Conclusions

Pedestrian activity recognition is a key issue in pedestrian navigation. Most previous studies have presented several solutions based on traditional methods, such as SVM, DT, and RF. In recent years, deep learning has developed rapidly. In this paper, we review previous studies on pedestrian activity recognition. Then, we provide the methodology of this study and present measurement preprocessing algorithms, including body acceleration extraction, de-noising, posture transformation, and FFT. We also present the whole transformation procedure of model training. Researchers can use Tensorflow to train deep learning models in their computers or servers. With the procedure, the model can be converted to .tflite mode, which can run in Android.

In this study, we designed deep learning models using the LSTM network and CNN. Four main experiments were performed. The first one was motion mode recognition, in which we used the UCI dataset and trained the experimental data with seven traditional ML classification methods and two designed deep learning models. The test results show LSTM and CNN had high accuracy with values of 90.74% and 91.92%, respectively. In the second experiment, we collected nine smartphone postures data using HUAWEI Mate20. We trained these data using the seven ML classification methods and the two deep learning methods. The test results show that LSTM and CNN both high accuracy. The constructed deep learning models had higher accuracy than the seven traditional ML classification methods; the values were 93.69% (LSTM) and 95.55% (CNN). In the third experiment, we designed five comprehensive activities that combined motion models and smartphone postures. The real-time test showed that the accuracy was up to 89.39%. Therefore, postures were recognized in a smartphone in real-time. In this study, although real-time pedestrian activity recognition was realized in an Android smartphone, the accuracy still needs to be improved, especially ‘Upstairs’ and ‘Downstairs’ are often recognized as other activities. In the future, we still need to research which features are useful to improve classification accuracy. Besides, deep learning models based on the LSTM network or CNN need to be improved, and the network structure of the training model needs to be optimized. In the last experiment, we verified the end navigation capability with activity recognition. The test results show that the scheme ‘PDR + GNSS + Beacon + Activity Recognition’ was improved. In the actual situation, the fusion process is complicated; we also need to research accurate navigation update algorithms for recognizing different pedestrian activities to improve positioning precision.

## Figures and Tables

**Table 1:** Features extracted from measurements.

Category	Type	Definition
Statistical	Mean	$\mathrm{mean}\left(u\right)=\bar{u[n]}=\frac{1}{N}\sum_{n=0}^{N-1}u\left[n\right]$
	Median	$\mathrm{median}\left(u\right)=\left\{\begin{array}{cc} u^{\prime }\left[\frac{N+1}{2}-1\right] & \;\mathrm{if}\;N\;\mathrm{is}\;\mathrm{odd}\;\\ \left.\frac{1}{2}\left([u^{\prime }\frac{N}{2}-1\right]+u^{\prime }\left[\frac{N}{2}\right]\right) & \;\mathrm{if}\;N\;\mathrm{is}\;\mathrm{even}\; \end{array}\right.$
	Root Mean Square	$\mathrm{rms}\left(u\right)=\sqrt{\bar{u^{2}\left[n\right]}}$
	75th percentile	percentile (u,75),$p=\frac{\;\mathrm{count}\;(u[n]<\;\mathrm{percentile}\;(u,p))}{N}\times 100$
	Variance	$\mathrm{var}\left(u\right)=\sigma_{u}^{2}=\bar{{\left(u-\bar{u}\right)}^{2}}$
	Standard Deviation	$\mathrm{std}\left(u\right)=\sigma_{u}$
	Skewness	$\mathrm{skew}\left(u\right)=\frac{\frac{1}{N}\sum_{n=0}^{N-1}{\left(u\left[n\right]-\bar{u}\right)}^{3}}{{\left(\sqrt{\frac{1}{N}\sum_{n=0}^{N-1}{\left(u\left[n\right]-\bar{u}\right)}^{2}}\right)}^{3}}$
	Binned Distribution	bindstbn (u,bin)=count(min(bin)≤u[n]<max(bin))
	Mean Absolute Deviation	$\mathrm{MAD}\left(u\right)=\bar{|u-\bar{u}|}$
Frequency Domain	Fourier Transform	$U\left(f_{k}\right)=\sum_{\forall n}u\left[n\right]e^{-j\frac{2\pi kn}{N}}$
	Short-Time Fourier Transform	$U\left(f_{k}\right)=\sum_{\forall n}u\left[n\right]e^{-j\frac{2\pi kn}{N}}$
	Discrete Cosine Transform	$\mathrm{STFT}\left(u\right)\left[k\right]=\sum_{\forall n}u\left[n\right]w\left[n\right]e^{-j\frac{2\pi kn}{N}}$
	Continuous Wavelet Transform	$\mathrm{DCT}\left(u\right)\left[k\right]=\sum_{n=0}^{N-1}u\left[n\right]\cos\left(\frac{\pi }{N}\left(n+\frac{1}{2}\right)k\right)$
	Discrete Wavelet Transform	$DWT_{\psi }\left(u\right)\left[m\right]=\frac{1}{\sqrt{2^{m}}}\sum_{vn}u\left[n\right]\psi \left(2^{-m}k-n\right)$
	Wigner Distribution	$\mathrm{WD}\left(u\right)\left[\omega \right]=\frac{1}{\sqrt{2\pi }}\int_{-\infty }^{\infty }u\left(t+\frac{\tau }{2}\right)u^{*}\left(t-\frac{\tau }{2}\right)e^{-j\omega \tau }d\tau$
	Frequency Domain Entropy	$H_{f}\left(u\left[n\right]\right)=\sum_{i=0}^{N}P_{i}\left(U\left(f_{i}\right)\right)\log\left(\frac{1}{P_{i}\left(U\left(f_{i}\right)\right)}\right)$
Energy, Power, Magnitude	Energy	energy $\left(u\right)=\sum_{n=0}^{N-1}{\left(u\left[n\right]\right)}^{2}$
	Sub-band Energies	energy${}_{l}\left(u\right)=\sum_{n=0}^{N-1}H_{BPF_{l}}^{2}\left(u\right)$
	Sub-band Energy Ratios	subband energy ${\mathrm{ratio}}_{i,j}\left(u\right)=\frac{\;\mathrm{subband}\;\mathrm{energy}{\;}_{i}\left(u\right)}{\;\mathrm{subband}\;\mathrm{energy}\;j}$
	Signal Magnitude Area	$\mathrm{SMA}\left(u\right)=\frac{1}{N}\sum_{n=0}^{N-1}\left|u\left[n\right]\right|$
Time Domain	Zero-Crossing Rate	$\mathrm{mean}\left(u\right)=\frac{1}{N-1}\sum_{n=0}^{N-1}I\left\{u\left[n\right]u\left[n-1\right]<0\right\}$

**Table 2:** Template of a confusion matrix for a four-class classifier.

Actual Mode	Predicted Mode			
	Class 1	Class 2	Class 3	Class 4
Class 1	$n_{11}$	$n_{12}$	$n_{13}$	$n_{14}$
Class 2	$n_{21}$	$n_{22}$	$n_{23}$	$n_{24}$
Class 3	$n_{31}$	$n_{32}$	$n_{33}$	$n_{34}$
Class 4	$n_{41}$	$n_{42}$	$n_{43}$	$n_{44}$

**Table 3:** Statistic information of prediction evaluation.

Measure	Description	Definition
True Positive	The number of samples of a class that are correctly classified	$TP_{i}=n_{ii}$
True Negative	The number of samples of other classes that are correctly classified	$TN_{i}=\sum_{j\neq i}\sum_{k\neq i}n_{jk}$
False Positive	The number of samples not belonging to a class that are incorrectly classified as belonging to it	$FP_{i}=\sum_{k\neq i}n_{ki}$
False Negative	The number of samples belonging to a class that are incorrectly classified as belonging to other class	$FN_{i}=\sum_{k\neq i}n_{ik}$
Recall	Proportion of cases of a class that are correctly classified	$TPR_{i}=\frac{TP_{i}}{P_{i}}=\frac{TP_{i}}{TP_{i}+FN_{i}}$
Accuracy	Proportion of all cases that are correctly classified	$ACC=\frac{\sum_{\forall i}n_{ii}}{n}$
Precision	Proportion of cases predicted to belong to a class that are correct	$PPV_{i}=\frac{TP_{i}}{TP_{i}+FP_{i}}$
F-Score	The weighted average of precision and sensitivity	$F1=\frac{2}{1/{\mathrm{Sens}}_{i}+1/{\mathrm{Prec}}_{i}}$
Sensitivity	The proportion of samples that are correctly classified	Sens ${}_{i}=\frac{TP_{i}}{TP_{i}+FP_{i}}$
AUC	The area under the curve (AUC) combines sensitivity and specificity, reflecting the overall performance of the classification model	refer to [44]
Specificity	The proportion of negative samples that are correctly classified to be negative	$Spec_{i}=\frac{TN_{i}}{FP_{i}+TN_{i}}$

**Table 4:** Pedestrian navigation update strategies.

Motion Mode	Navigation Update
Stationary	Fix 3D Position
	Apply ZUPT
Standing on Moving Walkway	Updated 2D Position
Walking	Apply PDR (Pedestrian Dead Reckon)
Walking on Moving Walkway	Increase 2D Displacement in Direction of Motion
	Apply PDR
Elevator	Fix 2D Position
	Update Altitude
Escalator Standing	Update 2D Position
	Update Altitude
Stairs	Project Displacement to Horizontal Plane
	Apply PDR
	Update Altitude
Escalator Walking	Increase 2D Displacement
	Project Displacement to Horizontal Plane
	Apply PDR
	Update Altitude

**Table 5:** Activity proportion of human activity recognition dataset of UCI.

STANDING	SITTING	LAYING	WALKING	DOWNSTAIRS	UPSTAIRS
1722	1544	1406	1777	1906	1944
16.72%	14.99%	13.65%	17.25%	18.51%	18.88%

**Table 6:** Classification results of seven traditional ML classification methods.

Model	AUC	Accuracy	F1-Score	Precision	Recall
SGD	0.664	0.446	0.427	0.419	0.446
Naive Bayes	0.734	0.736	0.747	0.734	0.880
DT	0.850	0.748	0.746	0.745	0.748
kNN	0.895	0.707	0.706	0.806	0.707
RF	0.966	0.818	0.818	0.819	0.818
NN	0.974	0.856	0.857	0.860	0.856
SVM	0.988	0.878	0.872	0.899	0.878

**Table 7:** Structure of the designed LSTM network model.

Layer (Type)	Output Shape	Parameter
LSTM	[(None, 128, 32)]	5376
LSTM	[(None, 128, 32)]	8320
LSTM	(None,32)	8320
Dropout	(None, 32)	0
Dense	(None, 6)	198

**Table 8:** Structure of designed CNN network model.

Layer (Type)	Output Shape	Param
Input Layer	[(None, 128, 9, 1)]	0
Conv2D	(None, 126, 7, 16)	160
Batch Normalisation	(None, 126, 7, 16)	64
Activation	(None, 126, 7, 16)	0
Conv2D	(None, 126, 7, 16)	2320
Batch Normalisation	(None, 126, 7, 16)	64
Activation	(None, 126, 7, 16)	0
MaxPooling2	(None, 63, 3, 16)	0
Conv2D	(None, 61, 1, 32)	4640
Batch Normalisation	(None, 61, 1, 32)	128
Activation	(None, 61, 1, 32)	0
Conv2D	(None, 61, 1, 32)	9248
Batch Normalisation	(None, 61, 1, 32)	128
Activation	(None, 61, 1, 32)	0
Flatten	(None, 1952)	0
Dense	(None, 128)	249,984
Batch Normalisation	(None, 128)	512
Activation	(None, 128)	0
Dropout	(None, 128)	0
Dense	(None, 6)	774

**Table 9:** Classification results using raw accelerometer, gyroscope, and magnetometer measurements.

Model	AUC	Accuracy	F1-Score	Precision	Recall
SGD	3.882	0.108	0.075	0.080	0.108
kNN	4.259	0.190	0.186	0.183	0.190
SVM	4.867	0.185	0.181	0.208	0.185
Naive Bayes	4.993	0.260	0.252	0.253	0.260
DT	4.660	0.310	0.307	0.308	0.310
RF	5.708	0.375	0.372	0.376	0.375
NN	6.454	0.481	0.478	0.478	0.481

**Table 10:** Classification results using raw accelerometer, gyroscope, magnetometer, and light measurements.

Model	AUC	Accuracy	F1-Score	Precision	Recall
SGD	4.263	0.195	0.141	0.149	0.195
kNN	4.565	0.251	0.225	0.236	0.251
SVM	4.920	0.222	0.226	0.273	0.222
Naive Bayes	5.959	0.304	0.291	0.290	0.304
DT	5.118	0.309	0.299	0.294	0.309
RF	5.928	0.370	0.363	0.359	0.370
NN	6.171	0.473	0.449	0.465	0.473

**Table 11:** Classification results using processed accelerometer, gyroscope, magnetometer, and light measurements.

Model	AUC	Accuracy	F1-Score	Precision	Recall
SGD	4.383	0.222	0.177	0.230	0.222
SVM	5.087	0.239	0.251	0.330	0.239
Naive Bayes	6.691	0.422	0.408	0.410	0.422
DT	6.160	0.601	0.599	0.603	0.601
kNN	6.499	0.647	0.639	0.671	0.647
RF	7.390	0.723	0.724	0.726	0.723
NN	7.046	0.722	0.714	0.747	0.722

**Table 12:** Classification results using processed accelerometer, gyroscope, and magnetometer measurements.

Model	AUC	Accuracy	F1-Score	Precision	Recall
SGD	4.034	0.143	0.114	0.197	0.143
SVM	5.548	0.253	0.249	0.324	0.253
Naive Bayes	6.027	0.344	0.338	0.337	0.344
DT	6.245	0.640	0.638	0.640	0.640
RF	7.233	0.703	0.701	0.703	0.703
NN	7.463	0.741	0.739	0.741	0.741
kNN	7.179	0.755	0.755	0.757	0.755

**Table 13:** Classification results using processed accelerometer, and gyroscope measurements.

Model	AUC	Accuracy	F1-Score	Precision	Recall
SGD	4.182	0.176	0.156	0.154	0.176
SVM	5.240	0.193	0.180	0.251	0.193
Naive Bayes	6.311	0.392	0.381	0.378	0.392
DT	6.672	0.729	0.729	0.730	0.729
kNN	7.227	0.770	0.770	0.773	0.770
RF	7.530	0.809	0.809	0.810	0.809
NN	7.608	0.814	0.814	0.816	0.814

**Table 14:** Evaluation results of the model trained by LSTM network.

	Accuracy	Precision	Recall	F1-Score Score
Test Results	90.74%	90.97%	90.74%	90.71%

**Table 15:** Evaluation results of the model trained by CNN network.

	Accuracy	Precision	Recall	F1-Score Score
Test Results	91.92%	92.79%	91.85%	91.77%

**Table 16:** Evaluation results of the model trained by LSTM network using raw measurements.

	Accuracy	Precision	Recall	F1-Score Score
Test Results	85.77%	85.67%	85.68%	85.66%

**Table 17:** Evaluation results of the model trained by LSTM network using filtered measurements.

	Accuracy	Precision	Recall	F1-Score Score
Test Results	93.69%	93.90%	93.69%	93.71%

**Table 18:** Evaluation results of the model trained by CNN network.

	Accuracy	Precision	Recall	F1-Score Score
Test Results	95.55%	96.04%	95.54%	95.63%

**Table 19:** Evaluation results of the model trained by CNN using measurements without light.

	Accuracy	Precision	Recall	F1-Score Score
Test Results	79.82%	79.82%	75.62%	78.35%

**Table 20:** Evaluation results of the model trained by CNN network using measurements with light.

	Accuracy	Precision	Recall	F1-Score Score
Test Results	89.39%	89.39%	87.15%	89.27%

## Abbreviations

The following abbreviations are used in this paper:

PDR	Pedestrian Dead Reckon
MEMS	Micro-electromechanical System Sensor
RNN	Recurrent Neural Network
CNN	Convolutional Neural Network
kNN	k-Nearest Neighbor
SGD	Stochastic Gradient Descent
SVM	Support Vector Machine
RF	Random Forest
NN	Neural Network
ANN	Artificial Neural Network
DCNN	Deep Convolutional Neural Network
DBN	Deep Belief Network
MLP	Multi-Layer Perceptron
ML	Machine Learning
GNSS	Global Navigation Satellite System
DT	Decision Tree
LSTM	Long Short-Term Memory
IOT	Internet of Things
